# Pediatric Residents’ Awareness and Practices Toward Critical Congenital Heart Disease Screening in Saudi Arabia: A Multicenter Study

**DOI:** 10.3390/ijns12010012

**Published:** 2026-02-27

**Authors:** Hussien Abdo Babiker, Turki Omaish Alotaibi, Hiba Hassan, Sulaiman Almohaimeed, Shadin Alamrah, Asalah Alhazmi, Abdulwahab H. Alharbi

**Affiliations:** 1 Pediatric and Neonatology Department, King Fahd Military Medical Complex, Dhahran 31932, Saudi Arabia; 2 Basic Medical Sciences Unit, Prince Sultan Military College of Health Sciences, Dhahran 31932, Saudi Arabia

**Keywords:** awareness, critical congenital heart disease, practice, pulse oximetry

## Abstract

Critical congenital heart disease (CCHD) is a major cause of neonatal morbidity and mortality. Pulse oximetry screening enables early detection, potentially reducing complications and improving outcomes. This study evaluated pediatric residents’ knowledge, attitudes, and practices (KAP) related to CCHD screening in Saudi Arabia. A cross-sectional survey was distributed to pediatric residents across Saudi Arabia. The questionnaire assessed knowledge, attitude, and practice regarding CCHD screening. A total of 123 pediatric residents in training were included in the study. Of these, 57 (46.3%) were male, and 66 (53.7%) were female. A progressive increase in mean scores was observed with advancing training years (*p* = 0.010). A significant difference was observed in knowledge scores based on completion of a cardiology rotation (*p* = 0.006). A progressive increase in attitude scores was observed with each successive year of training. Current year in training showed a statistically significant association with attitude scores (*p* < 0.001). Completion of a newborn nursery or NICU rotation was also significantly associated with higher attitude scores (*p* = 0.027). Similarly, attitude scores were significantly higher among those who had completed a cardiology rotation (mean = 12.99, SD = 1.52) compared to those who had not (mean = 11.60, SD = 1.84; *p* < 0.001). While practice scores were not statistically different across most groups, senior residents demonstrated better adherence to screening. Residents exhibit increasing awareness and positive attitudes with experience; however, practical implementation remains inconsistent. Targeted education and standardized protocols are necessary to improve outcomes. A positive correlation was observed between knowledge and attitude scores (r = 0.346, *p* < 0.001).

## 1. Introduction

Congenital Heart Disease (CHD) is one of the most common congenital anomalies and a major contributor to neonatal morbidity and mortality worldwide [1,2]. Globally, the incidence of CHD ranges from 4 to 50 per 1000 live births, with approximately 25% of these cases classified as Critical Congenital Heart Disease (CCHD)—a life-threatening subset requiring surgical or catheter-based intervention within the first year of life [2,3]. In Saudi Arabia (SA), the prevalence of CHD is estimated at approximately 14.8 per 1000 live births, aligning with international figures [4].

Delayed diagnosis of CCHD beyond hospital discharge significantly increases the risk of adverse outcomes. To address this, pulse oximetry screening has been widely adopted as a safe, sensitive, cost-effective, and non-invasive method for early detection of hypoxemia—a common indicator of CCHD [5,6,7]. This method is particularly effective in detecting conditions such as transposition of the great arteries and tetralogy of Fallot [8,9].

In response to global recommendations and mounting evidence, Saudi Arabia launched a nationwide pulse oximetry screening program in 2016, initially covering 30 hospitals, which expanded to 70 more in 2017, and achieved full national implementation by 2018 [6]. According to recent estimates, over 900,000 newborns have been screened in the Kingdom, with a CCHD detection rate of 0.26%, which is consistent with international benchmarks [2,10].

Despite the program’s wide implementation and evident success, its effectiveness hinges on the knowledge, attitudes, and practices (KAP) of frontline healthcare providers—particularly pediatric residents who are directly involved in neonatal care and early screening decisions. Prior studies in SA have highlighted gaps in awareness and inconsistent practices among pediatricians regarding neonatal screening protocols, including hearing and metabolic screenings. The study focused on pediatric residents since they are directly involved in frontline neonatal care, including initial evaluation and implementation of screening protocols, and because they are in a formative stage where training can have a significant impact on their future clinical practice.

Therefore, this study aims to assess the KAP of pediatric residents toward CCHD screening in Saudi Arabia, identify knowledge-practice gaps, and explore potential barriers to optimal implementation. The findings will inform training strategies and contribute to improving neonatal outcomes by enhancing the early detection of CCHD.

## 2. Materials and Methods

This study was approved by the Institutional Review Board (IRB) of Armed Forces Hospitals Eastern Province (AFHER), Dhahran (IRB Number AFHER-IRB-2024-33). All participants signed a written informed consent form.

For this cross-sectional study, a well-structured, validated, and pre-tested questionnaire was developed in accordance with the study’s objectives. The questionnaire was developed by a special research group following an extensive review of the literature. The questionnaire consisted of five sections: demographic variables, knowledge of critical congenital heart disease (5 questions), attitudes towards the screening protocol for these critical congenital heart diseases in Saudi Arabia (3 questions), and the practice score was measured using practical questions in accordance with the guidelines of the CCHD screening program implemented by the Ministry of Health, Saudi Arabia. The questionnaire also included open-ended questions for additional comments regarding CCHD screening practices and training.

Pediatric residents in training at different levels in Saudi Arabia were included in the study. Questions were validated through a panel of experts before conducting a pilot test of 48 participants, who were not included in the study. Cronbach’s alpha was used to assess the questionnaire’s internal consistency reliability; values of 0.7 indicated internal consistency reliability.

One score was allocated to each of the five multiple-choice knowledge and practice questions for each correct answer, totaling 5 points each. Wrong answers received no scores. The participants’ attitudes were assessed using a five-point Likert scale rating question: strongly agree (5), agree (4), neutral (3), disagree (2), and strongly disagree (1). The average attitude score was calculated out of 15 for the three attitude questions.

### 2.1. Study Design and Participants

A prospective cross-sectional study was conducted among a random sample of pediatric residents across Saudi Arabia. An anonymous, structured online questionnaire was distributed via institutional email lists and professional networks. Participation was voluntary.

### 2.2. Survey Instrument

The questionnaire consisted of five sections:

1. Demographics: Gender, training year, prior NICU and cardiology rotations.
2. Knowledge: Items based on national CCHD screening guidelines.
3. Attitude: Likert-scale items assessing perceived importance, confidence, and support for screening.
4. Practice: Self-reported adherence and perceived barriers.
5. Additional comments.

### 2.3. Statistical Analysis

Data were analyzed using SPSS software version 28.0 (SPSS, Chicago, IL, USA). Descriptive statistics summarized participant characteristics and KAP scores. Comparative analyses included *t*-tests and one-way ANOVA. Pearson correlation coefficients were calculated to assess associations between KAP domains. Statistical significance was set at *p* < 0.05.

## 3. Results

### 3.1. Characteristics of Pediatric Residents’ Study Participants

Table 1 shows the demographic and professional characteristics of the participant. A total of 123 randomly selected pediatric residents in training were included in the study. Of these, 57 (46.3%) were male, and 66 (53.7%) were female. Regarding the current year in training, the largest proportion of participants were in their first year (*n* = 40, 32.5%), followed by second-year trainees (*n* = 28, 22.8%). Fewer participants were in their third (*n* = 14, 11.4%), fourth (*n* = 24, 19.5%), and fifth year of training (*n* = 17, 13.8%). When asked about previous exposure to training on critical congenital heart disease (CCHD) screening, 47 participants (38.2%) reported prior exposure, while 76 (61.8%) reported no prior exposure. Most participants (*n* = 101, 82.1%) had completed a rotation in the newborn nursery or neonatal intensive care unit (NICU), whereas 22 (17.9%) had not. Additionally, 69 participants (56.6%) had completed a dedicated cardiology rotation, while 53 (43.4%) had not.

### 3.2. Knowledge

Table 2 shows the knowledge, attitude, and practice scores by demographic and professional characteristics of the participants. The mean knowledge scores were analyzed across various demographic and training characteristics. There was no statistically significant difference in knowledge scores between gender groups, with males scoring a mean of 3.6 (SD = 1.11) and females scoring 3.5 (SD = 0.96; *p* = 0.608). However, the current year in training was significantly associated with knowledge score (*p* = 0.010). First-year trainees had the lowest mean score at 3.15 (SD = 1.09), while fourth-year trainees had the highest at 4.04 (SD = 0.91). A progressive increase in mean scores was observed with advancing training years. Participants with prior exposure to critical congenital heart disease (CCHD) screening training had a higher mean knowledge score of 3.64 (SD = 1.05) compared to those without such exposure (mean = 3.49, SD = 1.03), although this difference was not statistically significant (*p* = 0.432).

Those who had completed a newborn nursery or NICU rotation had a higher mean knowledge score (3.59, SD = 1.05) than those who had not (3.32, SD = 0.95), but the difference was not statistically significant (*p* = 0.259).

A significant difference was observed in knowledge scores based on completion of a cardiology rotation (*p* = 0.006). Participants who had completed a cardiology rotation scored higher (mean = 3.75, SD = 0.93) compared to those who had not (mean = 3.25, SD = 1.09).

### 3.3. Attitude

Attitude scores were analyzed across demographic and clinical training variables (Table 2). Gender was not significantly associated with attitude scores; males had a mean score of 12.68 (SD = 1.69), and females had a mean of 12.17 (SD = 1.87; *p* = 0.113).

Current year in training showed a statistically significant association with attitude scores (*p* < 0.001). First-year trainees had the lowest mean score (11.50, SD = 1.73), whereas fifth-year trainees had the highest (13.41, SD = 1.58). A progressive increase in attitude scores was observed with each successive year of training.

Participants with prior exposure to critical congenital heart disease (CCHD) screening training had significantly higher attitude scores (mean = 13.32, SD = 1.48) than those without such exposure (mean = 11.84, SD = 1.77; *p* < 0.001). Completion of a newborn nursery or NICU rotation was also significantly associated with higher attitude scores (*p* = 0.027). Participants who had completed this rotation scored an average of 12.57 (SD = 1.77), compared to 11.64 (SD = 1.68) among those who had not.

Similarly, attitude scores were significantly higher among those who had completed a cardiology rotation (mean = 12.99, SD = 1.52) compared to those who had not (mean = 11.60, SD = 1.84; *p* < 0.001).

### 3.4. Practice

Practice scores, indicating the frequency with which participants performed critical congenital heart disease (CCHD) screenings, were compared across demographic and training variables (Table 2).

There was no statistically significant difference in practice scores between gender groups. Group 1 had a mean score of 3.60 (SD = 1.50), and Group 2 had a score of 3.15 (SD = 1.54; *p* = 0.442).

Practice scores did not significantly vary by year of training (*p* = 0.743). The highest mean scores were observed among third-year (4.07, SD = 1.27) and fifth-year trainees (3.88, SD = 1.41), while fourth-year trainees had the lowest score (2.96, SD = 1.52).

Participants with previous exposure to CCHD screening training had slightly higher mean practice scores (3.66, SD = 1.46) than those without such exposure (3.17, SD = 1.55), though the difference was not statistically significant (*p* = 0.507).

Similarly, no significant differences were observed based on completion of a newborn nursery/NICU rotation (*p* = 0.990). Those who had completed the rotation scored an average of 3.38 (SD = 1.54), compared to 3.27 (SD = 1.55) among those who had not.

Participants who had completed a cardiology rotation also showed higher mean practice scores (3.49, SD = 1.46) than those who had not (3.15, SD = 1.61), but the difference was not statistically significant (*p* = 0.344).

### 3.5. Follow-Up Actions After Failed CCHD Screening

Table 3 presents the follow-up practice scores, receipt of formal training on CCHD screening, and perceived barriers to CCHD screening, categorized by the demographic and professional characteristics of the participants. Practice scores related to appropriate follow-up actions after a failed CCHD screening were analyzed in relation to demographic and clinical training characteristics.

There was no significant difference in follow-up practice scores by gender (Group 1: mean = 1.19, SD = 0.40; Group 2: mean = 1.21, SD = 0.41; *p* = 0.793).

However, a statistically significant difference was observed across training years (*p* < 0.001). Fifth-year trainees reported the highest mean score (1.65, SD = 0.49), suggesting more consistent or appropriate follow-up practices. In contrast, first- through fourth-year trainees had comparable lower scores ranging from 1.11 to 1.15.

Participants with prior CCHD screening training had slightly higher follow-up scores (mean = 1.26, SD = 0.44) than those without (mean = 1.17, SD = 0.38), though this was not statistically significant (*p* = 0.259). Similarly, those who completed a newborn nursery or NICU rotation had higher scores (mean = 1.23, SD = 0.42) compared to those who had not (mean = 1.09, SD = 0.29; *p* = 0.148), but the difference was not statistically significant.

Participants who had completed a cardiology rotation had higher follow-up scores (mean = 1.23, SD = 0.43) compared to those who had not (mean = 1.15, SD = 0.36; *p* = 0.265).

### 3.6. Receipt of Formal Training on CCHD Screening

Responses regarding whether participants had received formal training on critical congenital heart disease (CCHD) screening were compared across demographic and training characteristics (Table 3). Scores ranged from 1 = “No” to 2 = “Yes,” with higher scores indicating receipt of formal training.

There was no significant difference in responses between genders (Male: mean = 1.88, SD = 0.33; Female: mean = 1.91, SD = 0.29; *p* = 0.566).

Year of training was significantly associated with receiving formal CCHD training (*p* < 0.001). Fifth-year trainees reported the lowest average score (1.59, SD = 0.51), suggesting that fewer of them received formal training. In contrast, first-year trainees had the highest average score (1.98, SD = 0.16), indicating they were more likely to have received recent formal instruction.

Participants with prior exposure to CCHD screening training were significantly more likely to report having received formal training (mean = 1.97, SD = 0.16) compared to those without such exposure (mean = 1.77, SD = 0.43; *p* < 0.001).

Participants who had completed a cardiology rotation were significantly less likely to report receiving formal training than those who had not (1.83 vs. 1.98; *p* = 0.006).

Completion of a newborn nursery/NICU rotation showed a non-significant trend toward fewer reports of formal training (*p* = 0.075), although all respondents without the rotation uniformly reported receiving training (mean = 2.00, SD = 0.00).

Results of Perceived Need for Additional Training or Resources on CCHD Screening

Participants’ perceptions regarding whether they would benefit from additional training or resources on CCHD screening were evaluated across demographic and clinical training variables. Responses were coded on a binary scale, with 1 = “Yes” and 2 = “No”; thus, a mean closer to 1 indicates a greater perceived need.

There were no statistically significant differences in perceived need for further training by gender (Group 1: mean = 1.09, SD = 0.29; Group 2: mean = 1.09, SD = 0.29; *p* = 0.951).

By year of training, perceived need was generally consistent, ranging from 1.04 to 1.18, with no significant association detected (*p* = 0.398). Fifth-year trainees reported the highest perceived need (mean = 1.18, SD = 0.39), while second- and fourth-year trainees reported the lowest (mean = 1.04).

There was no significant difference between those who had received previous exposure to CCHD screening training and those who had not (means = 1.09 vs. 1.09; *p* = 0.895).

Similarly, perceived need did not significantly vary by newborn nursery/NICU rotation (*p* = 0.395) or by cardiology rotation (*p* = 0.619).

Overall, participants across all groups expressed a consistently high perceived need for additional training, with mean values very close to 1.0.

### 3.7. Correlation Between Knowledge, Attitude, and Practice Scores

Table 4 shows the correlation analysis between knowledge, attitude, and practice scores toward CCHD screening in Saudi Arabia. Knowledge and attitude show a moderate, statistically significant positive correlation. On the other hand, attitude and practice show a weak to moderate, statistically significant positive correlation. However, knowledge and practice show no statistically significant correlation.

### 3.8. Perceived Barriers to Implementing CCHD Screening)

Participants rated perceived barriers to implementing critical congenital heart disease (CCHD) screening on a scale, with higher mean scores indicating a greater perception of barriers (Table 3). The analysis examined the relationships between barrier scores and demographic/training characteristics. There was a non-significant trend toward higher perceived barriers among males (mean = 1.82, SD = 1.20) compared to females (mean = 1.33, SD = 0.79; *p* = 0.067).

Barrier perception increased slightly with advanced years of training, from a mean of 1.42 (SD = 0.81) in first-year trainees to 1.82 (SD = 1.13) in fifth-year trainees, though the association was not statistically significant (*p* = 0.317).

Participants with prior exposure to CCHD screening training had a higher mean barrier score (1.79, SD = 1.20) than those without (1.42, SD = 0.88), but this difference was also not statistically significant (*p* = 0.268).

Trainees who had completed a newborn nursery or NICU rotation reported slightly higher perceived barriers (mean = 1.60, SD = 1.09) compared to those without that experience (mean = 1.36, SD = 0.66; *p* = 0.073).

Similarly, participants who had completed a cardiology rotation had higher mean barrier scores (1.64, SD = 1.14) than those who had not (1.43, SD = 0.84), although this difference did not reach statistical significance (*p* = 0.122).

Overall, perceived barriers were moderately low across groups, with some variability depending on gender and clinical exposure.

## 4. Discussion

Congenital heart disease (CHD) remains a significant cause of neonatal morbidity and mortality worldwide, and among these, critical congenital heart disease (CCHD) poses the greatest risk if undetected. CCHD includes life-threatening cardiac malformations that typically require surgical or catheter-based intervention in the first year of life. Early detection is crucial for preventing hemodynamic compromise and improving outcomes [1,11].

Pediatric residency training in Saudi Arabia is strong, yet it differs in terms of clinical experiences and educational focus among institutions. Although national CCHD screening policies exist, residency programs would benefit from uniform educational approaches to guarantee that all trainees attain proficiency in the implementation and interpretation of screening. Integrating CCHD screening into established curricula, evaluations, and quality enhancement efforts, while coordinating training with national screening data systems, can enhance clinician preparedness and lead to better neonatal cardiovascular results in Saudi pediatric training programs [12].

In our study, we assessed the knowledge, attitudes, and practices of pediatric residents regarding CCHD screening in Saudi Arabia, and we evaluated the clinical outcomes associated with early pulse oximetry screening. Our findings underscore both the importance and feasibility of implementing standardized pulse oximetry screening for all newborns prior to hospital discharge.

Our results support the existing literature, which demonstrates the utility of pulse oximetry in detecting CCHD in asymptomatic newborns. Ewer et al. screened 20,055 newborns using pulse oximetry and identified 53 cases of major CHD, including 24 with CCHD, yielding a prevalence of 2.6 per 1000 live births. The reported sensitivity and specificity of the screening were satisfactory [13]. These findings are consistent with those of Thangaratinam et al., who in a meta-analysis showed that pulse oximetry is a reliable, non-invasive tool with high specificity and moderate sensitivity for CCHD detection [3].

The timing of diagnosis significantly influences both preoperative and postoperative outcomes in neonates with CCHD. Multiple studies have confirmed that early identification reduces the incidence of circulatory collapse, improves stability before surgery, and enhances survival [14,15].

Delayed diagnosis is associated with increased risk of acidosis, organ damage, and death. Our study findings underscore the necessity of early screening and timely referral for echocardiographic confirmation.

Systematic screening for CCHD with high accuracy requires next-generation pulse oximeters capable of detecting subtle hypoxemia. Additionally, comparing oxygen saturation levels between the right hand (pre-ductal) and one foot (post-ductal) significantly improves the detection of duct-dependent lesions [16,17]. This dual-site approach helps identify differential cyanosis, a key indicator of certain types of CCHD, and has become an essential part of contemporary screening protocols.

Kemper et al. developed national strategies to implement CCHD screening in the United States, concluding that a standardized approach involving pulse oximetry prior to discharge was both practical and cost-effective [10]. Their work has influenced the global adoption of CCHD screening protocols and inspired similar initiatives in Europe and the Middle East.

Despite its advantages, pulse oximetry has limitations. It may not detect a certain cyanotic or left-sided obstructive lesion (e.g., coarctation of the aorta or interrupted aortic arch). Moreover, false-positive results can occur in cases of pulmonary disease or delayed transition [8]. Thus, positive screens must always be followed by echocardiographic evaluation and consultation with pediatric cardiology.

To date, data from Saudi Arabia regarding CCHD screening remains limited. However, our findings align with previous reports showing a high prevalence of CHD in Saudi newborns and the urgent need for early screening strategies [6,8]. National implementation of pulse oximetry screening programs can bridge the gap between birth and timely cardiac diagnosis, reducing preventable neonatal deaths and improving the quality of life for affected families [11].

The current findings suggest a moderate and statistically significant positive correlation between knowledge and attitude toward critical congenital heart disease screening. This indicates that higher knowledge scores are associated with more favorable attitudes. In contrast, the lack of a significant correlation between knowledge and practice implies that knowledge alone may not be sufficient to translate into actual screening behaviors. The weak to moderate association between attitude and practice highlights the influence of personal beliefs and motivation on the implementation of these practices. Therefore, interventions should focus not only on improving knowledge but also on strengthening positive attitudes to enhance screening practices [12].

Healthcare provider training, adequate resource allocation, and public health education campaigns are essential to the successful implementation of these initiatives. Given the high burden of CHD in the region, integrating pulse oximetry into routine newborn care is both a logical and necessary step.

The limitations of this study are that the conclusions drawn from descriptive and exploratory items are not inferential.

## 5. Conclusions

Early identification of critical congenital heart disease (CCHD) is essential for improving neonatal survival and long-term outcomes. Our findings support the use of routine pulse oximetry screening as an effective, non-invasive, and low-cost method for early detection of CCHD. Pediatric residents demonstrated moderate knowledge and favorable attitudes toward implementing such screening, indicating a positive environment for adopting national protocols. Comparing oxygen saturation between the right hand and foot substantially enhances detection accuracy and should be incorporated into all screening algorithms. National-level implementation of standardized screening protocols can significantly reduce diagnostic delays and improve health outcomes for neonates in Saudi Arabia.

## Figures and Tables

**Table 1:** Demographic and professional characteristics of the participant (*n* = 123).

Variable		Frequency (n)	Percentage
Gender	Male	57	46.3
	Female	66	53.7
Current year of training	Year 1	40	32.5
	Year 2	28	22.8
	Year 3	14	11.4
	Year 4	24	19.5
	Year 5	17	13.8
CCHD Training Exposure	Exposed	47	38.2
	Not exposed	76	61.8
Newborn nursery/NICU rotation	Completed	101	82.1
	Not completed	22	17.9
Cardiology rotation	Completed	69	56.6
	Not completed	53	43.3

**Table 2:** Knowledge, attitude, and practice scores by demographic and professional characteristics of the participant (*n* = 123).

		Knowledge Out of 5		Attitude Out of 15		Practice Out of 5	
Variable	Group	Mean ± SD	*p*-Value	Mean ± SD	*p*-Value	Mean ± SD	*p*-Value
Gender	Male	3.6 ± 1.11	0.608	12.68 ± 1.69	0.113	3.60 ± 1.50	0.442
	Female	3.5 ± 0.96		12.17 ± 1.87		3.15 ± 1.54	
Current Year in Training	Year 1	3.15 ± 1.09	0.010	11.50 ± 1.73	<0.001	3.20 ± 1.56	0.743
	Year 2	3.46 ± 0.96		12.11 ± 1.71		3.25 ± 1.60	
	Year 3	3.64 ± 0.75		12.93 ± 1.82		4.07 ± 1.27	
	Year 4	4.04 ± 0.91		13.25 ± 1.42		2.96 ± 1.52	
	Year 5	3.82 ± 1.07		13.41 ± 1.58		3.88 ± 1.41	
CCHD Training Exposure	Exposed	3.64 ± 1.05	0.432	13.32 ± 1.48	<0.001	3.66 ± 1.46	0.507
	Not Exposed	3.49 ± 1.03		11.84 ± 1.77		3.17 ± 1.55	
Newborn Nursery/NICU Rotation	Completed	3.59 ± 1.05	0.259	12.57 ± 1.77	0.027	3.38 ± 1.54	0.990
	Not completed	3.32 ± 0.95		11.64 ± 1.68		3.27 ± 1.55	
Cardiology Rotation Completed	Completed	3.75 ± 0.93	0.006	12.99 ± 1.52	<0.001	3.49 ± 1.46	0.344
	Not completed	3.25 ± 1.09		11.60 ± 1.84		3.15 ± 1.61	

**Table 3:** Follow-up practice scores, receipt of formal training on CCHD screening, and perceived barriers to CCHD screening by demographic and professional characteristics of the participant (*n* = 123).

		Follow-Up Practice Scores Out of 2		Receipt of Formal Training on CCHD Screening Out of 4		Perceived Barriers to CCHD Screening Out of 2	
Variable	Group	Mean ± SD	*p*-Value	Mean ± SD	*p*-Value	Mean ± SD	*p*-Value
Gender	Male	1.19 ± 0.40	0.793	1.88 ± 0.33	0.566	1.82 ± 1.20	0.067
	Female	1.21 ± 0.41		1.91 ± 0.29		1.33 ± 0.79	
Current Year in Training	Year 1	1.15 ± 0.36	<0.001	1.98 ± 0.16	<0.001	1.42 ± 0.81	0.317
	Year 2	1.11 ± 0.32		1.96 ± 0.19		1.57 ± 1.10	
	Year 3	1.14 ± 0.36		1.93 ± 0.27		1.36 ± 0.93	
	Year 4	1.13 ± 0.34		1.87 ± 0.34		1.71 ± 1.23	
	Year 5	1.65 ± 0.49		1.59 ± 0.51		1.82 ± 1.13	
CCHD Training Exposure	Exposed	1.26 ± 0.44	0.259	1.77 ± 0.43	<0.001	1.79 ± 1.20	0.268
	Not exposed	1.17 ± 0.38		1.97 ± 0.16		1.42 ± 0.88	
Newborn Nursery/NICU Rotation	Completed	1.23 ± 0.42	0.148	1.87 ± 0.34	0.075	1.60 ± 1.09	0.073
	Not completed	1.09 ± 0.29		2.00 ± 0.00		1.36 ± 0.66	
Cardiology Rotation Completed	Completed	1.23 ± 0.43	0.265	1.83 ± 0.38		1.64 ± 1.14	0.122
	Not completed	1.15 ± 0.36		1.98 ± 0.14		1.43 ± 0.84	

**Table 4:** Pearson correlation analysis between knowledge, attitude, and practice scores toward critical congenital heart disease screening in Saudi Arabia (*n* = 123).

Measured Variable	Pearson Correlation	*p*-Value
Knowledge—Attitude	0.346	<0.001
Knowledge—Practice	0.047	0.605
Attitude—Practice	0.291	0.001

## Data Availability

The data are not publicly available due to privacy and ethical restrictions but are available from the corresponding author upon reasonable request and with permission from the relevant institutional review board.

## Abbreviations

The following abbreviations are used in this manuscript:

CHD	Congenital Heart Disease
CCHD	Critical Congenital Heart Disease
KAP	Knowledge Attitude and Practice
SD	Standard Deviation
IRB	Institutional Review Board.
AFHER	Armed Forces Hospitals Eastern Region
NICU	Neonatal Intensive Care Unit
